# An Auditory Illusion of Infinite Tempo Change Based on Multiple Temporal Levels

**DOI:** 10.1371/journal.pone.0008151

**Published:** 2009-12-03

**Authors:** Guy Madison

**Affiliations:** Department of Psychology, Umeå University, Umeå, Sweden; CNRS, France

## Abstract

Humans and a few select insect and reptile species synchronise inter-individual behaviour without any time lag by predicting the time of future events rather than reacting to them. This is evident in music performance, dance, and drill. Although repetition of equal time intervals (i.e. isochrony) is the central principle for such prediction, this simple information is used in a flexible and complex way that accommodates both multiples, subdivisions, and gradual changes of intervals. The scope of this flexibility remains largely uncharted, and the underlying mechanisms are a matter for speculation. Here I report an auditory illusion that highlights some aspects of this behaviour and that provides a powerful tool for its future study. A sound pattern is described that affords multiple alternative and concurrent rates of recurrence (temporal levels). An algorithm that systematically controls time intervals and the relative loudness among these levels creates an illusion that the perceived rate speeds up or slows down infinitely. Human participants synchronised hand movements with their perceived rate of events, and exhibited a change in their movement rate that was several times larger than the physical change in the sound pattern. The illusion demonstrates the duality between the external signal and the internal predictive process, such that people's tendency to follow their own subjective pulse overrides the overall properties of the stimulus pattern. Furthermore, accurate synchronisation with sounds separated by more than 8 s demonstrate that multiple temporal levels are employed for facilitating temporal organisation and integration by the human brain. A number of applications of the illusion and the stimulus pattern are suggested.

## Introduction

Periodical sound patterns such as rhythmic music induce a sensation of temporal recurrence called pulse [1]. This is employed by humans for achieving precise synchronisation or entrainment among individuals in music performance, dance, drill, and various ritual behaviours [2]. Pulse is a periodic process that enables proactive timing by predicting the time of future events rather than reacting to them with a time lag of at least 100 ms [3]. This makes precise synchronisation and co-ordination among individuals possible in the absence of any other common control system [4]. While pulse events typically coincide with conspicuous physical events in the sensory signal, such as louder or more frequent sounds, the pulse is in fact a subjective process. Pulse events may occur at points in time when there is no physical stimulus, if implied by the stimulus pattern as a whole [5], as well as in the absence of an external signal altogether [6].

Music is the most common example of a sound pattern with multiple temporal levels, in contrast to which the typical signal of a metronome can be characterised as a one-level pattern that physically contains only one level at which to attribute pulse. Even in response to one-level patterns may the pulse be perceived at other levels than the physical one, determined in part by temporal limits of the neural system [7], [8]. This so-called subjective rhythmisation [9] is typically attributed to multiples corresponding to every second, third, or fourth sound etcetera if the physical intervals are short, and to subdivisions of intervals into two, three, or more equal subintervals if the physical intervals are long. Although repetition of equal time intervals (i.e. isochrony) is the central principle for predictive timing, this simple information is apparently used in a flexible and complex way that accommodates not only fractions and subdivisions of intervals, but also momentary [1] and gradual [10] changes of intervals. The scope of this flexibility remains largely uncharted, and the underlying mechanisms are a matter for speculation [e.g. 11], [12], [13], [14].

Temporal synchronisation among individuals is rare in the animal kingdom, which indicates that is has few instrumental uses. In addition to humans it is nevertheless found in a few species of insects and reptiles, for example, for which its function is to increase the salience or geographic reach of a signal by summation over simultaneously signalling individuals. This is in turn used for attracting migrating females [15], [16], warning conspecifics for predators [17], or confusing the auditory localising ability of bats preying on a species of tree frog [18], for example. The underlying mechanisms are in general poorly understood, although the synchronising behaviour of fireflies has been subject to quite detailed study [19]–[21]. These examples show that the ability and motivation for entrainment is not merely a curiosity but may serve a distinct adaptive function whenever its function has been identified. For humans, however, no obvious adaptive function has been recognised for entrainment, although it is obviously a key element in music performance and other group behaviours mentioned above. While neither these behaviours are unanimously attributed to an adaptive value, music and entrainment in various guises has been suggested to be related to group cohesion [22], the evolution of language [23], hominid speciation by means of natural selection [24], and costly signalling of mate quality in the context of sexual selection [25]. Be that as it may; human entrainment capabilities serve important functions in humans' present state of affairs, and have long attracted scientific inquiry [e.g.], [4], [26,27].

**Figure 1:**
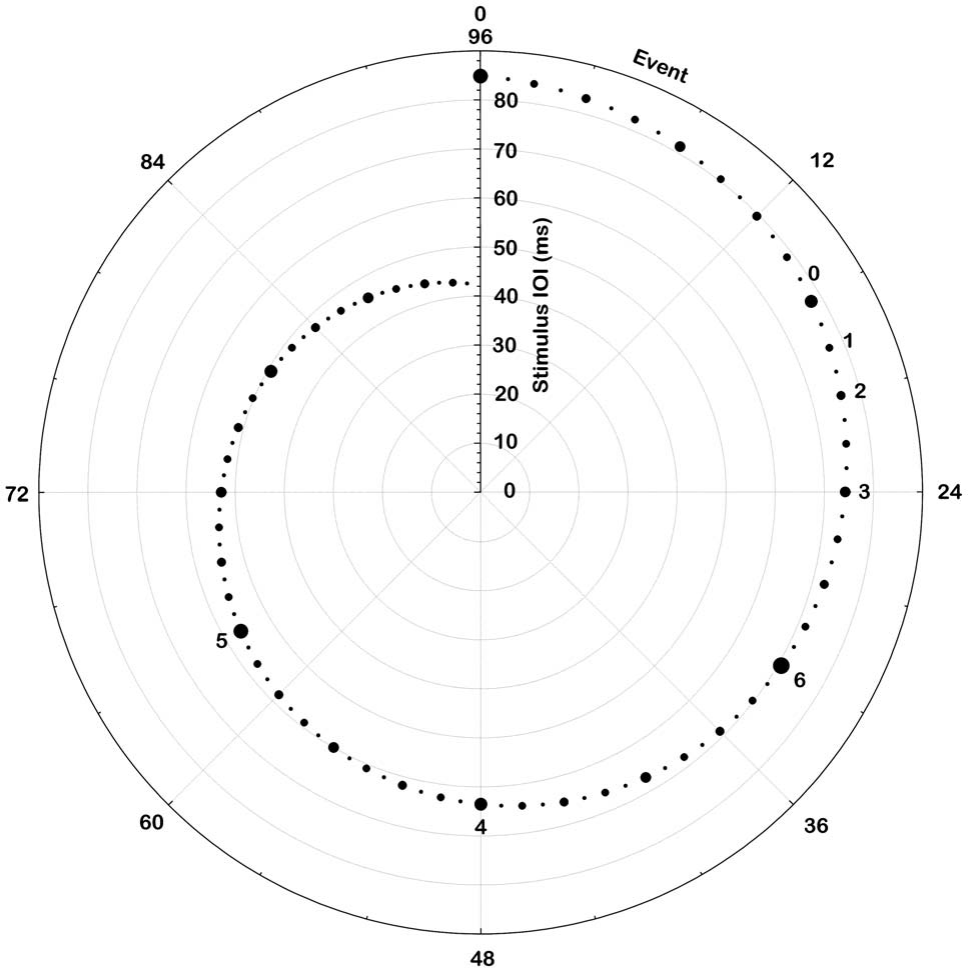
Implementation of the multiple temporal levels in a 96-event example pattern.

Given that human processing of isochronous sequences accommodates multiplication and subdivision of intervals it is not surprising that music is also characterised by this multiple temporal level structure, as defined by metre and different note values. The present illusion is based on a generalisation of this property. While the presence of each level typically varies throughout a piece of real music, the present pattern features physical events corresponding to all levels within a perceptually relevant range. The pattern consists of sounds with brief isochronous inter onset intervals (IOI) on the order of 50 ms. Every second sound event is louder than the intervening ones, which gives the impression of a second temporal level with half the rate of the first one. Increasing the loudness of every second event of the second level likewise yields a third level, and so forth. In addition to this, the intervals are continuously increased or decreased by a factor of 2^±1^ across the pattern length, that is, either halving or doubling the interval. These principle properties are illustrated in Figure 1, which for clarity of presentation depicts an example 96-event pattern rather than the 786-event patterns used in the experiment. The ordinal position of sound events in the pattern is indicated by the angular scale, and their IOI by the radial scale. The size of the points represents the relative loudness of events. It is manipulated so as to obscure the boundaries between pattern repetitions, which might otherwise distract listeners and make the illusion less powerful (cf. Shepard's circularity in the perception of pitch illusion [28]). All these features are detailed in Materials and Methods.

It was predicted that participants would accurately synchronise with one level in the pattern and follow the gradual interval change therein, but would continue to the next higher or lower level at pattern boundaries, depending on the direction of the change. This illusion was evaluated by a sensori-motor synchronisation experiment in which human participants were asked to beat a drumstick against an electronic sensor plate in synchrony with their perceived pulse.

## Results and Discussion

**Figure 2:**
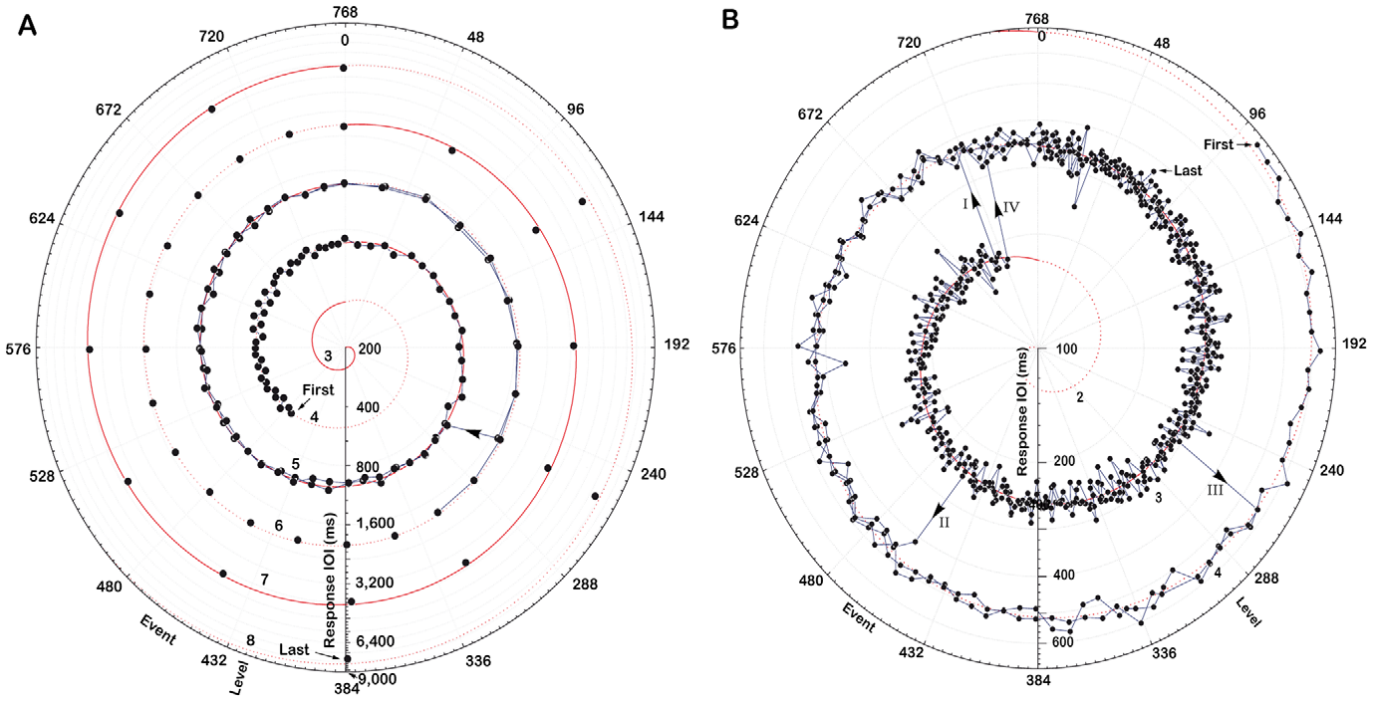
Polar representations of two response sequences.

Responses demonstrated epochs of consistent synchronisation with sounds corresponding to one specific level, seamless continuation to an adjacent level at the pattern boundary, and occasional switching to another level when beat IOIs become very short or long. All these features occurred in almost every trial (N = 76), and are examplified by one trial with increasing intervals in Figure 2A (Audio file S2) and one trial with decreasing intervals in Figure 2B (Audio file S4). The course of each example trial is represented by a clockwise trajectory with its first and last response intervals indicated, in Figure 2A for a trial with increasing intervals from the center (short intervals) to the circumference (long intervals), and in Figure 2B from the circumference to the center. Filled circles denote each response according to its closest corresponding event position in the stimulus pattern on the angular scale and to its IOI on the radial scale. When trajectories overlap (i.e., when synchronization to the same level has occurred more than once in the same trial) points are connected with lines to guide the eye. One response sequence corresponds to five revolutions, since the whole stimulus sequence in each trial consisted of five seamlessly repeated patterns. The illusory temporal levels that emerge as an effect of the manipulation of stimulus IOI and loudness are represented by the helical alternating dotted and solid lines numbered with Arabic numerals, whose meeting points coincide with the boundaries between repeated patterns at the zenit of the figure. Since the angular scale represents pattern position, and the time between successive events continuosly changes by a factor 2^±1^ across one pattern, time proper is nonlinear along this scale. This is evident at the boundaries between repeated patterns, where the distance between points to the right of the boundary is double the number of pattern events of the immediately preceding points to the left, although they in fact are separated by almost the same amount of time. The lines with arrows between levels indicate switches in the response interval, consecutively numbered with Roman numerals. Note that the stimulus pattern depicted in Figure 1 cannot be seen in these diagrams because it would be outside the radial scale (below 100 ms).

As can be seen, no switches coincide with the boundary between repeated patterns, which was indeed rarely the case among all trials. This suggests that participants did neither notice the boundary between pattern repetitions nor find any portion of the pattern more or less difficult to synchronise with. When interviewed after the session did no participant report having noticed any breakpoints in the stimulus sequence. On the contrary, several participants spontaneously reported being puzzled by making such large changes in beat rate without the stimulus essentially changing. To quantify this issue, the 351 switches that occurred across all 76 trials were sorted in 16 bins according to their pattern position; one bin for each 48 adjacent positions of the total 768. No significant difference in the frequency of switches was found among bins (χ^2^ = 13.64, *df* = 15, *p* = 0.55), a histogram of which showed no tendency for frequencies to increase close to the start or end of the pattern (Figure S1).

Within these features of the response sequences, demonstrating the illusion, the beat IOI varied among trials and participants in accordance with the unconstrained task. Participants did accurately synchronise with IOIs longer than 8 s (Fig. 2A) and shorter than 200 ms (Fig. 2B), while many trials exhibited a more narrow range. Across all trials, central tendencies in IOI for switches were close to 0.5 s for decreasing IOIs (M = 511, Md = 461, min = 160, max = 1070 ms) and close to 2 s for increasing IOIs (M = 2197, Md = 1778, min = 713, max = 8650 ms).

**Figure 3:**
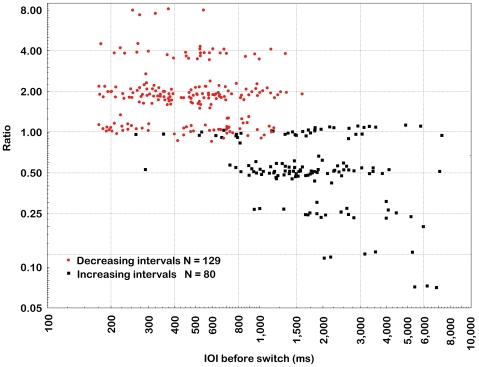
Ratios of switch inter onset intervals (IOI) as a function of beat interval before the switch. See text for further explanations.

Most aspects of the switching behaviour are summarised in Figure 3, in which the ratio between the last IOI before the switch and the first IOI after the switch is plotted as a function of IOI before the switch. To render a clearer depiction of the results only switches with a relatively high effect size (Hedges' g>3.0) are included (N = 209) in order to exclude switches to a similar IOI (ratio ∼1), and axis scales are logarithmic. First, decreasing intervals (circles) lead to short IOIs that tend to be switched to the double IOI (2.0), while increasing intervals (squares) lead to long IOIs that tend to be switched to half or ¼ of the interval (0.5 or 0.25), as exemplified in Figure 2. Second, more extreme IOIs tend to be associated with larger switch ratios, in other words a tendency towards a preferred range of IOIs close to 500 ms [29]. This is particularly evident for increasing intervals, where IOIs above 4 s are exclusively divided in four at the switch. Third, switch IOIs along the abscissa for decreasing intervals suggest a bimodal distribution with central tendencies close to 300 and 600 ms, respectively, which is also reflected by the difference between the mean (511 ms) and the median (461 ms). This indicates the employment of two different strategies on behalf of the participants; either to switch within the comfortable range of IOIs between 400 and 800 ms [30], or to hold on until approaching the motor limit close to 200 ms [3].

The illusion was also demonstrated by the differences between increasing and decreasing sequences in both response and switch IOIs, which are - in contrast to the 2^±1^ stimulus change - on average on the order of 2^±2^. The central tendencies are 2197/511 = 4.30 for the mean IOI and 1778/461 = 3.86 for for the median IOI. It should be noted that this behaviour occurred spontaneously to the open-ended instruction to beat in synchrony with the perceived pulse and feel free to change the beat rate to a more natural one at any time. The mean change would conceivably have been much larger, in accord with the behaviour of some participants, if the instruction would have been to maintain the “same“ rate, which in the case of this pattern sequence would amount to following the illusory change for even longer epochs. Indeed, switches from one level to another were interspersed among long epochs subject to the illusion, and the actually produced IOIs therefore covered an even wider range than did the switch IOIs. In particular did a number of the response sequences with increasing intervals exhibit few or no switches at all, as exemplified by Figure 2A. All 19 participants produced beat sequences with local mean IOIs longer than 2 s, and many participants produced IOIs longer than 3 s (N = 17), 5 s (N = 11), and 8 s (N = 6).

Illusions offer insight into the working principles of perceptual and cognitive systems. Apart from the illusory phenomenon per se, their unstability and sensitivity to subtle stimulus properties can be employed for examining more general questions that may be difficult to address with normal stimuli. There are numerous visual illusions but only a handful auditory ones [31]. Shepard tones [28] and the present infinite tempo change create the only “impossible“ percepts while other auditory illusions involve hearing what is not physically present or choosing among ambiguous percepts (but cf. [32], and references therein).

The present data provide a striking demonstration of pulse and its subjective nature, characteristic for the proactive, predictive, and hypothesis-testing character of brain function in general [33], [34]. Specifically, the beat rate is apparently a function of both the immediate stimulus properties, which determine the possible specific time points to synchronise with bottom-up, and the recent behaviour history, which determines the level to synchronise with top-down. In other words, the pulse seems to function as a top-down hypothesis about future intervals, which assimilates contradictory sensory information if the discrepancy is not too large, but accommodates information that consistently disagrees with the hypothesis. In this case the ±0.13 percent (1/768) continuous stimulus interval change was readily accommodated into the prediction that the next interval was almost similar to the previous one, rather than determined by the local stimulus properties. It has previously been demonstrated that people can accurately synchronize with sound events across a wide range of change from ±0.077 to ±0.67 percent per interval in one-level sequences [10]. When local variability is applied to a stimulus sequence, in terms of unpredictable lengthening and shortening of otherwise isochronous intervals, the mean threshold for perceiving pulse was 8.6 percent across a group of listeners, as compared to a 3.5 percent threshold for detecting such deviations [1]. Although such ample margins may seem to suggest that synchronisation is correspondingly inexact, this is not the case. When deviations are relatively small, they are reflected in the immediately following response interval even when they are below the detection threshold [35], [36], although the proportion of the deviation reflected seems to decrease for larger (10–50%) deviations [37]. This seems to reflect another strategy for facilitating synchronisation, namely to rely more on the internal pulse when the signal is a poor predictor, but follow the signal closely when it is a reliable predictor of isochrony [e.g., 38]. The signal might be anything from rhythmic music or a multilevel pattern to the movements of another individual. There is evidence that the pulse mechanism is in place already within the first year of life [39].

The present results demonstrate proactive synchronisation, and hence temporal integration, of events separated by up to 8 s, while proactive synchronisation is impossible for one-level sequences with IOIs from 2.4 s and up [8]. Participants must therefore have utilised sound events intervening the level of their beats, at least for beat IOIs longer than 2 s. This demonstrates that although the pulse takes on a dominating role for the top-down interpretation of ambiguous stimuli, it does not preclude the influence of other information. These trade-off characteristics and their underlying mechanisms are a challenge for future research. Extant synchronisation models cannot account for this duality since they are confined to one level [e.g. 12], [40], [41]. Interestingly, however, both the present behaviours and type of stimuli are sufficiently well-defined to allow precise formal manipulation and modelling, and may therefore serve as a micro-world system for exploring general information-processing principles in ecologic, dynamical interaction.

The multilevel pattern could be applied to examine a number of issues that have been difficult to assess with traditional methods. It features a large and multi-dimensional stimulus space including base IOI, direction, rate of change, and the number of pattern repetitions. In the present study both directions were used but only one level of rate change (1/768≈0.13% per interval). In addition, sound events belonging to specific levels in the multilevel structure can be individually varied in time, loudness or excluded altogether, and their sound properties altered. Finally, all these aspects can be manipulated in the course of stimulus presentation, thereby adapting to a multitude of design requirements including dynamical, on-line change of parameters. For example, manipulating the relative sound levels in a multilevel structure makes it possible to vary perceived event rate while keeping the overall density of perceived events constant, which may be particularly useful for keeping activity related to sensory processing constant in brain imaging experiments. More generally, multilevel patterns may be more ecologically valid than the metronome-like clicks widely used both in basic research and clinical assessment [42]. They are likely to provide a more motivating stimulus by virtue of recruiting larger neural networks, in analogy with so-called rippled sound profiles [43] that comprise both a wide harmonic spectrum (analoguous to the multiple levels) and pitch sweeping (analoguous to the rate change). This property might be particularly valuable for use with children and clinical populations such as ADHD, who may be less able to focus sustained attention to demanding laboratory tasks.

## Materials and Methods

### Ethics Statement

All participants signed a written informed consent, were treated in accordance with the Declaration of Helsinki, and received a reward equivalent to USD 10. The study protocol was approved by the local ethics research committee at the University of Uppsala.

### Stimuli

The principle features of the illusive stimulus sequence are illustrated in Figures 1 and S2. Figure S2A shows the first 68 events of the multilevel structure. The temporal level (which basically controls loudness in the sound pattern) is shown on the ordinate, as a function of position in the pattern on the abscissa: every other event corresponds to level 0 (2^0^), every second event to level 1 (2^1^), and so forth. Only levels up to 6 fit within the range of the abscissa, so levels 7 (2^7^ =  every 128th event) and 8 (2^8^ = 256th event) are not shown.

Four different stimulus sequences were used. Each sequence had either increasing or decreasing intervals and a base IOI (mean IOI for level 0) of either 49 or 64 ms. They are available in mp3 format as Audio file S1 (increasing IOIs with 49 ms base IOI) Audio file S2 (increasing IOIs, 64 ms), Audio file S3 (decreasing, 49 ms), and Audio file S4 (decreasing, 64 ms).

The stimulus sequences were implemented with a pattern consisting of 768 events numbered 0–767, in which each event was assigned to one of 9 temporal levels according to 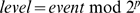, 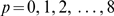. The IOI from each stimulus event to the next one was computed as 
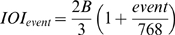
, where B is the base IOI. Two base IOIs were used in order to further assess possible effects of pattern position on the salience of the perceived pulse. The fraction between the two base IOIs (49 and 64 ms) was 1.30, which was chosen so as to be unharmonic with the inherent 2^±1^ relation and its aggregates in the multilevel pattern (e.g., 3/2 = 1.5). This means that the same IOIs occurred at pattern positions separated by 30 percent of the pattern length across patterns with different base IOI. The mean switch IOIs were not affected by base IOI, neither for decreasing (t = 1.19, df = 295, p = .55) nor increasing intervals (t = 1.44, df = 145, p = .59), which indicates that perceived rate was unaffected by pattern position per se.

The equations yield sound patterns in which the first and last IOIs were ∼32.67 and ∼65.29 ms for 49 ms base IOI and ∼42.67 and ∼85.28 for 64 ms base IOI. Expected pulse IOIs at pattern boundaries were powers of 2 within the manageable range for synchronisation, e.g. 261, 523, 1045, and 2091 ms for 49 ms base IOI and 341, 683, and 1365 ms for 64 ms base IOI. Each stimulus sequence consisted of 5 identical patterns presented in succession, which means that the maximum possible change in pulse rate was a factor of 32 (2^5^). Each sequence comprised 3840 events and a total duration of 188.16 s for 49 ms base IOI or 245.76 s for 64 ms base IOI.

Loudness was controlled by the note velocity parameter in the MIDI protocol [44], which can assume values between 0 and 127, and was computed as 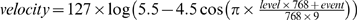. Loudness was further transformed in both hardware and software for adaptation to the hearing range. As these particulars are specific for the equipment used, the actual sound pressure level (SPL) in the headphones are recorded in a table that can be obtained on request. Essentially, SPL for the fastest level varied from 35 and 39 dBA, which encompassed the auditory detection threshold under the actual circumstances, and the SPL for the slowest level was ∼79 dBA. The room where the experiment took place had a 36 dBA background noise, mainly from the ventilation system, that was reduced to 31 dBA in the headphones. Figure S2B illustrates the loudness manipulation by means of a short example with increasing intervals (slowing rate). This example is only 96 events in order to convey the principles withouth cluttering the graph, but is computed with the same IOI and velocity equations as were the 768-event stimulus patterns used in the experiment. Increasing IOIs between sound events appear as longer distances between events from left to right, since the abscissa represents real time. The ordinate represents the loudness manipulation, showing that (a) the loudness of each level at the beginning of the pattern corresponds with an adjacent level at the end of the pattern, (b) loudness is a sigmoid function of level, such that it changes less from start to end for the fastest and slowest levels than for intermediate levels, (c) loudness is divided among all levels such that the fastest level transgresses the auditory threshold within the pattern, which masks its appearance (for increasing IOIs) or disappearance (for decreasing IOIs) at the pattern boundary. These devices are similar to those applied in Roger Shepard's circularity in pitch illusion [28].

### Apparatus

A PC with the real-time operating system FreeDOS was programmed to generate stimulus events and collect responses by means of an *MPU-401*–compatible MIDI interface connected to an Alesis DM5 synthesiser module. The temporal resolution of this system is 1 ms [see, e.g., 45]. Stimuli consisted of a percussion instrument sound (Prc/021 ShakerLo) presented through sound-attenuating Peltor HTB7A headphones. Responses were obtained by beating a drumstick against a *ddrum* drum pad equipped with a piezoelectric sensor (Clavia musical instruments, Stockholm).

### Participants

Nineteen nonmusicians participated, ten women and nine men aged 19 to 35 years (M = 24.5). None had any previous experience with similar experiments, had received music tuition, or had played a musical intrument in a systematic fashion.

### Procedure

Each individual session lasted 45–55 minutes and comprised instructions and 5 brief training trials together with the experimenter, followed by 28 static (without rate change) trials, 4 rate change trials, and then another 26 static trials performed alone. The static trials were not considered here. A critical part of the instruction was to beat the drum pad in synchrony with the perceived pulse, and to feel free to change the beat rate to a more natural one at any time.

### Data Analysis

Data consisted of IOIs of response beats and the identity (level and postion) of the pattern events closest in time to each beat. One important aspect of the participants' behaviour was at what positions in the stimulus sequence they switched from one beat rate to another. To detect switches from one consistent beat IOI to another one in an objective fashion, a routine based on Hedges' effect size *g* was implemented in Statistica v. 7 visual basic (Statsoft Inc.). It computes local means and variances and compares them for each position in order to find local maxima. For each response beat interval sequence X, M_1_ and s_1_^2^ were computed for X_i_ to X_i+9_, and M_2_ and s_2_^2^ were computed for X_i+10_ to X_i+20_. The effect size of the difference between these means was computed as 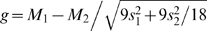. The difference M_1_–M_2_ was also multiplied with direction (-1 for decreasing intervals and 1 for increasing intervals) which excluded switches in the “wrong” direction. The initial *g*_1_ was stored as maximum *g* (*g*_max_) and the position i advanced one step. Subsequent larger values of *g* replaced *g*_max_ until *g*_i_<g_max_, which indicated that *g*_i-1_ was a local maximum and that a switch to a different mean IOI had occurred. If in these cases *g* was greater than a lower cut-off of 0.3, the sequence position and the mean IOIs before and after this position were stored, *g*_max_ was reset to 0, and the process re-continued. This procedure yielded 606 switches within the total 26,850 response intervals. However, some of these referred to switches in IOI within the same level in the pattern, and were therefore not relevant for testing possible correspondence between switch positions and pattern boundaries. Analyses showed that a cut-off of g>1.4 yielded switches (N = 351) that mostly referred to different levels in the pattern, i.e. differences with a factor 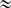 2^±1^ or greater.

## Supporting Information

Figure S1 Histogram of sequence positions at which the 351 switches with g>1.4 occurred.
(0.34 MB TIF)

Figure S2 Additional graphical representations of the stimulus pattern. See text for explanations.
(0.09 MB TIF)

Audio S1 Increasing intervals with 49 ms mean IOI
(3.09 MB MP3)

Audio S2 Increasing intervals with 64 ms mean IOI
(3.88 MB MP3)

Audio S3 Decreasing intervals with 49 ms mean IOI
(3.11 MB MP3)

Audio S4 Decreasing intervals with 64 ms mean IOI
(3.84 MB MP3)
