# Supplementary figures and images for: An Auditory Illusion of Infinite Tempo Change Based on Multiple Temporal Levels
Source: PLoS One. 2009 Dec 3;4(12):e8151. doi: 10.1371/journal.pone.0008151 (PMC2780720; doi:10.1371/journal.pone.0008151)

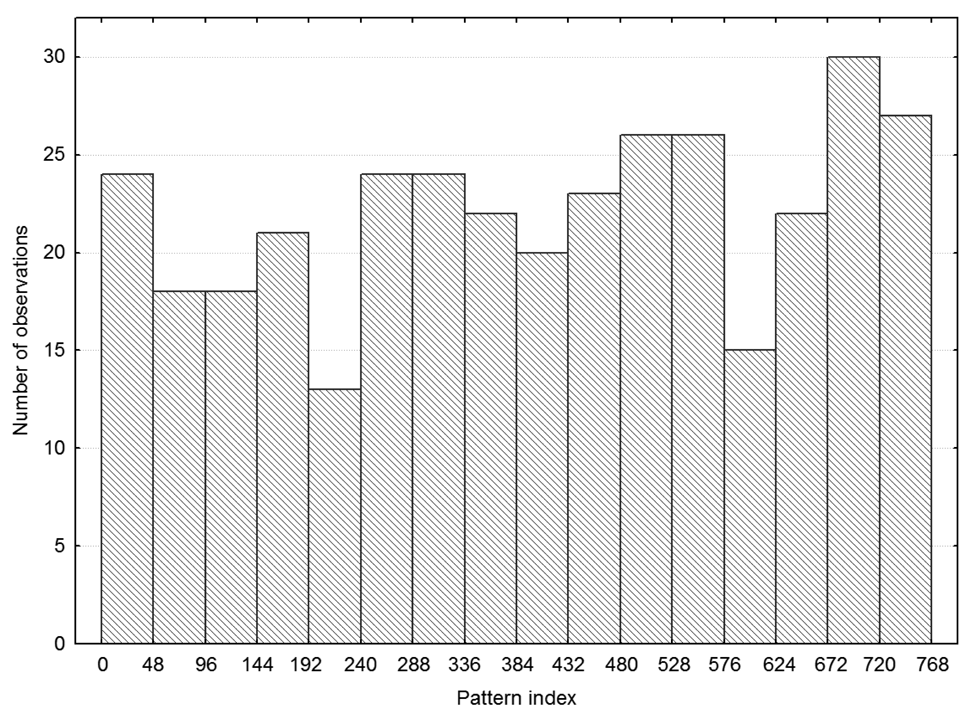

Supplement: Figure S1 — Histogram of sequence positions at which the 351 switches with g>1.4 occurred. (0.34 MB TIF) [file pone.0008151.s001.tif]

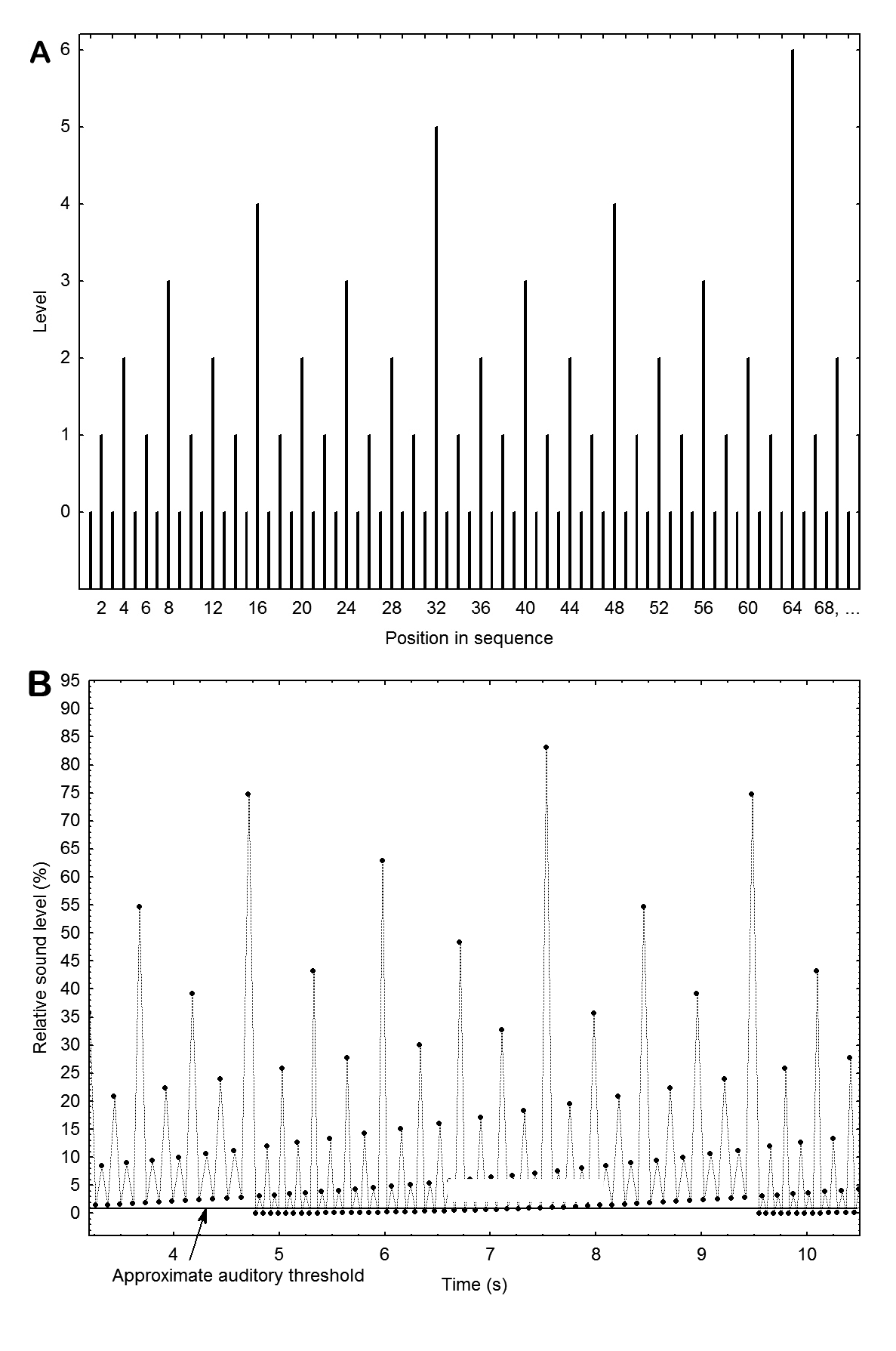

Supplement: Figure S2 — Additional graphical representations of the stimulus pattern. See text for explanations. (0.09 MB TIF) [file pone.0008151.s002.tif]
